# Dual direction CRISPR transcriptional regulation screening uncovers gene networks driving drug resistance

**DOI:** 10.1038/s41598-017-18172-6

**Published:** 2017-12-18

**Authors:** Carlos le Sage, Steffen Lawo, Prince Panicker, Tim M. E. Scales, Syed Asad Rahman, Annette S. Little, Nicola J. McCarthy, Jonathan D. Moore, Benedict C. S. Cross

**Affiliations:** Horizon Discovery, 8100 Cambridge Research Park, Waterbeach, Cambridge CB25 9TL United Kingdom

## Abstract

Pooled CRISPR–Cas9 knock out screens provide a valuable addition to the methods available for novel drug target identification and validation. However, where gene editing is targeted to amplified loci, the resulting multiple DNA cleavage events can be a cause of false positive hit identification. The generation of nuclease deficient versions of Cas9 has enabled the development of two additional techniques – CRISPR interference (CRISPRi) and CRISPR activation (CRISPRa) – that enable the repression or overexpression, respectively, of target genes. Here we report the first direct combination of all three approaches (CRISPRko, CRISPRi and CRISPRa) in the context of genome-wide screens to identify components that influence resistance and sensitivity to the BRAF inhibitor, vemurafenib. The pairing of both loss- and gain-of-function datasets reveals complex gene networks which control drug response and illustrates how such data can add substantial confidence to target identification and validation analyses.

## Introduction

The CRISPR (Clustered Regularly Interspaced Short Palindromic Repeats) system forms part of an adaptive immune response in bacteria^1,2^ and components of this complex have been co-opted to provide remarkably adaptable and accurate tools for engineering and animal model generation and in functional genomic studies^3^. When applied to pooled genetic screening, these tools provide new opportunities for drug target identification and validation. Cas9 when expressed with a suitably designed sgRNA molecule induces DNA double strand breaks which are repaired endogenously, principally by non-homologous end joining (NHEJ) which results in the introduction of insertions or deletions (InDels) and potential disruption of the locus of interest^4^. CRISPR-based screens enable the simultaneous evaluation of knocking out thousands of individual genes by coupling the analysis to next generation sequencing (NGS). Multiple experimental paradigms have been successfully explored with this technology, exploring both cell survival and in biomarker-linked screens using high-throughput flow cytometry^5^ and with several approaches to data analysis in each case, including algorithms such as MAGeCK^6^ and BAGEL^7^.

As the use of these CRISPR knock out (CRISPRko) screens has increased, some of the limitations of this approach have become apparent, such as genes within an amplified region of the genome scoring as false positives in synthetic lethal screens^8–10^. One way round this issue, which can be substantial in screens carried out in cancer cell lines owing to their often-disrupted genome, is to use the recently developed approaches of CRISPR interference (CRISPRi) and CRISPR activation (CRISPRa). These techniques make use of a nuclease-dead version of Cas9 (dCas9). Rather than mutating specific genomic loci, dCas9 allows disruption of gene transcription by the binding to proximal sequences at or near the transcriptional start site (TSS) of the gene. CRISPRi can efficiently block transcript initiation in *E. coli* and mammalian cells^11–15^, and this is substantially improved by covalently linking a KRAB transcriptional repressor to dCas9^16^. A similar approach is used in CRISPRa through the fusion of VP64 and p65 activation domains to dCas9^15,17–19^. Effective gene activation with CRISPRa has been driven by multiple adapted systems: the SunTag array, which uses multiple VP64s recruited onto a peptide array^20^; VPR, a tripartite activation method using a fusion of VP64, p65 and Rta^21^; and the Synergistic Activation Mediator complex (SAM^22^), which uses a dCas9-VP64 and recruitment of p65 and HSF1 via RNA binding protein components. These adapted CRISPR tools provide new opportunities in functional genomics and have been successfully deployed in pooled gain-of-function and loss-of-function genome-wide screens^16,22^.

For dCas9 to achieve full functionality, it has to exert its activating or inhibitory effect on a gene’s output through constitutive binding. Hinz *et al*.^23^, Horlbeck *et al*.^24^, and Isaac *et al*.^25^, discovered that dCas9 activity is influenced by nucleosome occupancy and that histone-DNA binding effectively blocks guideRNA access^23,25,26^. By coupling data available from a multitude of CRISPRi and CRISPRa screens to transcription start-site analysis (FANTOM), Horlbeck *et al*.^24^ were able to generate highly optimised versions of their guideRNA design platforms. This has substantially increased the performance of these tools. With these latest modifications in mind, we set out to compare the CRISPRko, CRISPRi and CRISPRa outputs in the context of a drug resistance screen.

We have conducted whole genome CRISPRko screening using 6 sgRNAs per target gene as described by the Zhang laboratory^27^ to identify genes that when lost give rise to resistance to the standard of care drug vemurafenib and to validate the results of a published screen^28^. To validate the use of CRISPRi and CRISPRa for pooled genetic screens, we explored this same clinical paradigm to allow us to directly compare data sets in the same cell line with the same drug concentration over the same time course of drug exposure. The CRISPRa vectors designed by the Zhang laboratory had also been evaluated against another BRAF inhibitor PLX-4720, giving an additional published resource of comparison^22^.

Our data clearly show that all three approaches, CRISPRko, CRISPRi and CRISPRa identify the same genes as driving resistance to vemurafinib. However, our data also indicate that CRISPRi is potentially a more sensitive approach compared with CRISPRko and that CRISPRi can also detect genes that induce drug sensitivity when knocked down. A comparison between CRISPRi and CRISPRa shows a clear concordance between gene loss driving resistance and these sgRNAs being selected against in CRISPRa screens, and vice versa. Thus, the employment of both CRISPRi and CRISPRa screens can provide greater confidence in targets identified in genome wide CRISPR screens.

## Results

**Figure 1:**
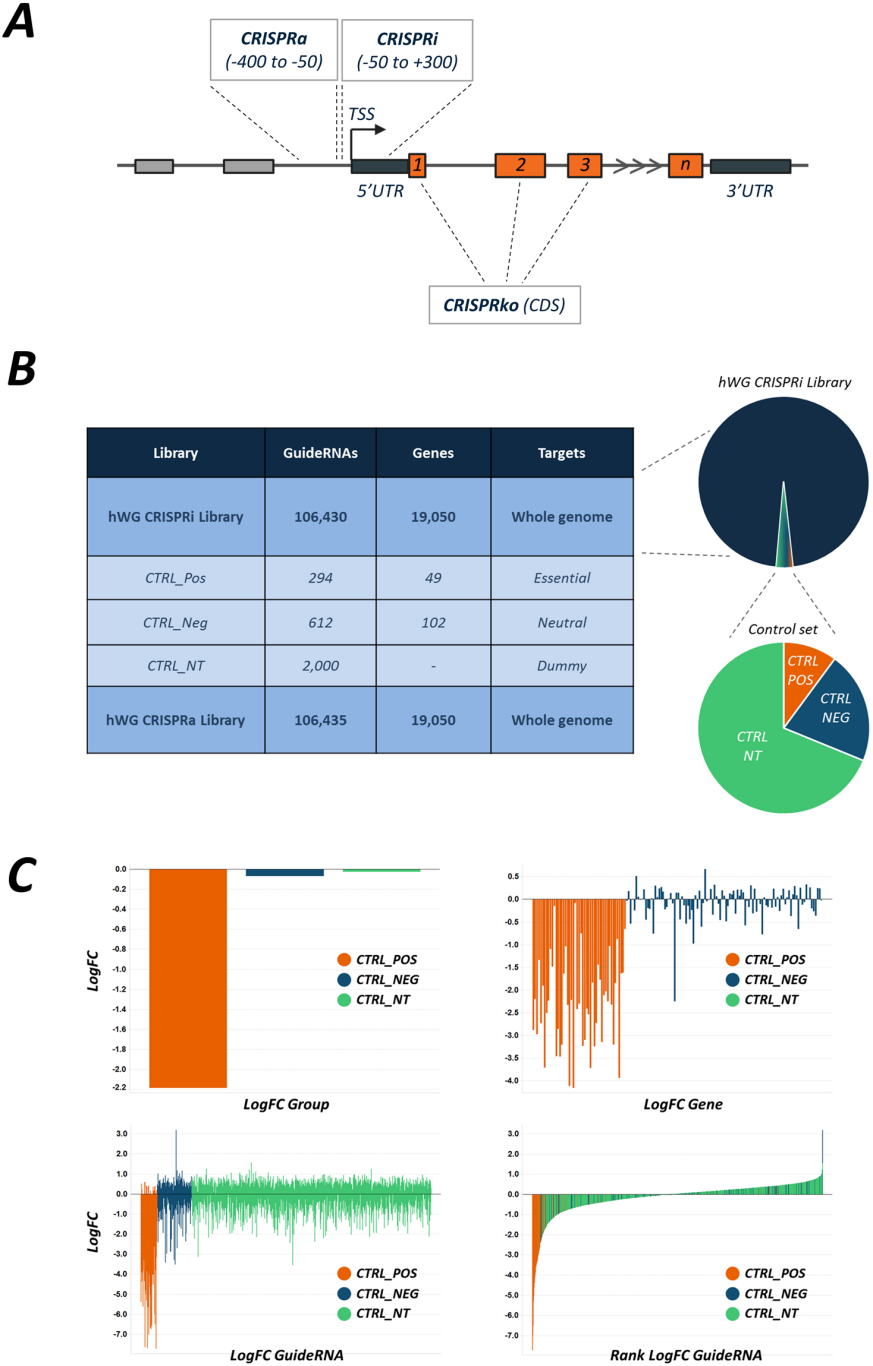
(**A**) Schematic showing location of guide targeting for each of CRISPRko, CRISPRi and CRISPRa. (**B**) Table and illustration of the constituents of the whole genome libraries and the nominated control groups. (**C**) Control groups behaviour. Top left show the mean log fold change between the input sample (plasmid baseline) and end point control (DMSO sample) for the groups of controls. Top right shows the gene-level mean data for each of the control genes. Bottom panels shows the individual guide-level data either segregated by group (left) or in a waterfall plot by value (right), indicating the partitioning of the positive control in the highly active group.

**Figure 2:**
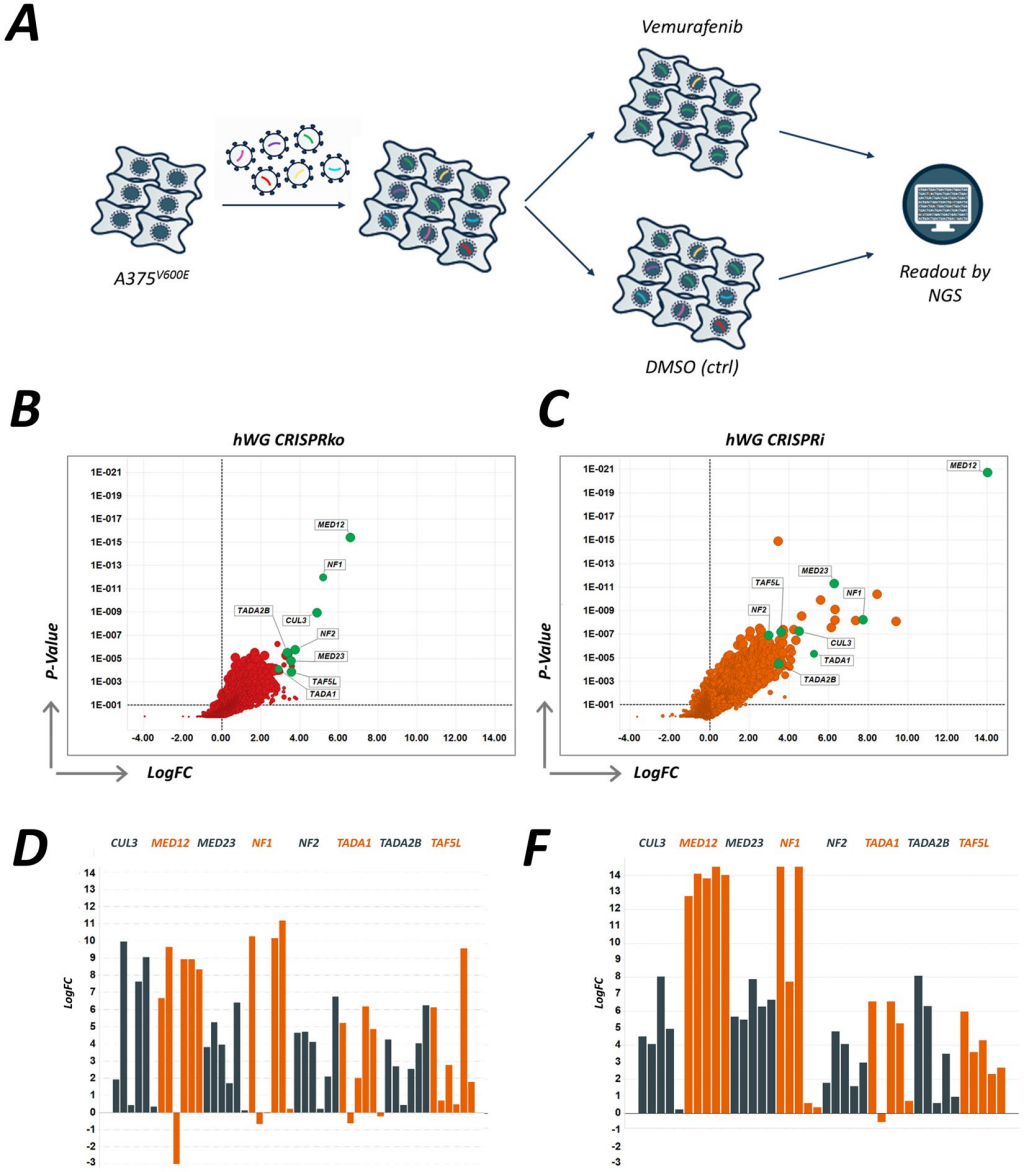
(**A**) Screen design and schematic. (**B**) Screen results from CRISPRko resistance screen, plotting the magnitude of the effect (LogFC) against the MAGeCK RRA score (p-value) and highlighting eight key hits (DMSO vs. vemurafenib). (**C**) Results from the CRISPRi resistance screen with overlapping hits from the CRISPRko screen highlighted. (**D**) Log2 enrichment scores for each of the individual guides from hit genes in the CRISPRko screen. (**E**) Log2 enrichment scores for each of the individual guides from hit genes in the CRISPRi screen.

We generated two pooled guide RNA whole-genome libraries using the algorithm described by Horlbeck *et al*.^24^ (Fig. 1A and Figure S4). The constituents of these libraries are shown in Fig. 1B. For CRISPRi, we developed and used a novel single vector system that expresses both dCas9–KRAB and sgRNAs and for CRISPRa we used a three vector system as initially described by Konermann *et al*.^22^. For both systems, lentiviral particles were generated using HEK293T cells. The hypotriploid A375 melanoma cell line was transduced with each library and selected using appropriate antibiotics. After 3 days of antibiotic selection, the antibiotic was removed and cells were split into 2 technical replicates and exposed to 2 μM of vemurafinib or to DMSO and the cells were grown for 16 population doublings (Fig. 2A).

Our initial QC analysis indicated that the control guides (positive, negative and dummy guides) in the CRISPRi library performed as anticipated (Fig. 1C). Comparison of the CRISPRi data with the CRISPRko data indicates a robust overlap of potential hits (Fig. 2B and C, and Fig. 2D and E). The most abundant hits are members of the Mediator complex, with the most enriched hit being MED12, a subunit involved in activation and repression of transcription. As with a previously published vemurafinib screen^27^ and our in-house CRISPRko data, members of the Mediator complex score highly in the CRISPRi screen, as do TAF6L, TAF5L, CCDC101, TADA1 and TADA2B, all of which are members of the SPT3–TAF(II)31–GCN5L acetylase (STAGA) complex, which has functions in chromatin modification, transcription, splicing and DNA repair machineries. Neurofibromin 1 (NF1), a negative regulator of RAS signalling, and NF2, a critical regulator of contact-dependent inhibition of proliferation which functions at the interface between cell-cell adhesion, transmembrane signalling and the actin cytoskeleton, also score well in both screens. In addition to the substantial overlap between the hits identified by the CRISPRko and CRISPRi screens, CRISPRi identified additional genes that could lead to vemurafenib resistance (Table 1).

**Table 1:** CRISPRi hits for vemurafenib resistance.

GENE	GUIDES	RRA Score	p-value	FDR	RANK	Good guides	LogFC
*MED12*	5	1.86E-21	2.64E-07	0.00045	1	5	14.015
*FOXD3*	10	1.27E-15	2.64E-07	0.00045	2	10	3.4554
*MED23*	5	5.12E-12	2.64E-07	0.00045	3	5	6.2753
*SUPT20H*	5	4.07E-11	2.64E-07	0.00045	4	5	8.4439
*CAND1*	5	1.22E-10	2.64E-07	0.00045	5	5	5.5962
*CCDC101*	5	7.67E-10	2.64E-07	0.00045	6	5	6.3151
*MED16*	5	2.93E-09	2.64E-07	0.00045	7	5	4.6355
*NF1*	5	5.67E-09	2.64E-07	0.00045	8	5	7.7366
*PGD*	5	6.44E-09	2.64E-07	0.00045	9	5	6.3138
*MED24*	5	6.89E-09	2.64E-07	0.00045	10	5	7.3586
*SMARCE1*	5	7.69E-09	2.64E-07	0.00045	11	5	9.4007
*TAF6L*	5	2.60E-08	7.91E-07	0.000646	12	5	6.1438
*MED15*	10	3.16E-08	7.91E-07	0.000646	13	7	2.5277
*HSD17B10*	5	4.03E-08	7.91E-07	0.000646	14	5	3.7132
*SOX10*	5	4.07E-08	7.91E-07	0.000646	15	5	4.2445
*ATP5J2*	5	4.90E-08	7.91E-07	0.000646	16	5	2.5933
*TTI2*	5	5.22E-08	7.91E-07	0.000646	17	5	3.7147
*CUL3*	5	5.45E-08	7.91E-07	0.000646	18	5	4.5056
*CUL5*	5	5.54E-08	7.91E-07	0.000646	19	5	2.0333
*CCNC*	5	6.15E-08	7.91E-07	0.000646	20	5	2.581
*TAF5L*	5	6.58E-08	7.91E-07	0.000646	21	5	3.5924
*NF2*	5	1.19E-07	7.91E-07	0.000646	22	5	2.9668
*NARS2*	5	1.27E-07	7.91E-07	0.000646	23	5	3.7081
*KIRREL*	5	1.51E-07	1.32E-06	0.000917	24	5	2.0196
*CSNK2B*	5	1.83E-07	1.32E-06	0.000917	25	5	2.6521

Interestingly, CRISPRi appears to be a more sensitive technique than CRISPRko, indicated by the substantially improved detection for MED12 and MED23 in both screens. Some of this increase in sensitivity is likely to be due to the use of an improved tracrRNA sequence^29^ in the CRISPRi vector, which was not used in the CRISPRko screen (Figure S1). At the guide level, the increased sensitivity for both MED12 and MED23 might also be attributable to the fact that all 5 guides are active and therefore substantially enriched in the screen, whereas for other potential hits, such as NF1, fewer guides appear to be active (Fig. 2D). However, these genes still clearly score as significant hits.

**Figure 3:**
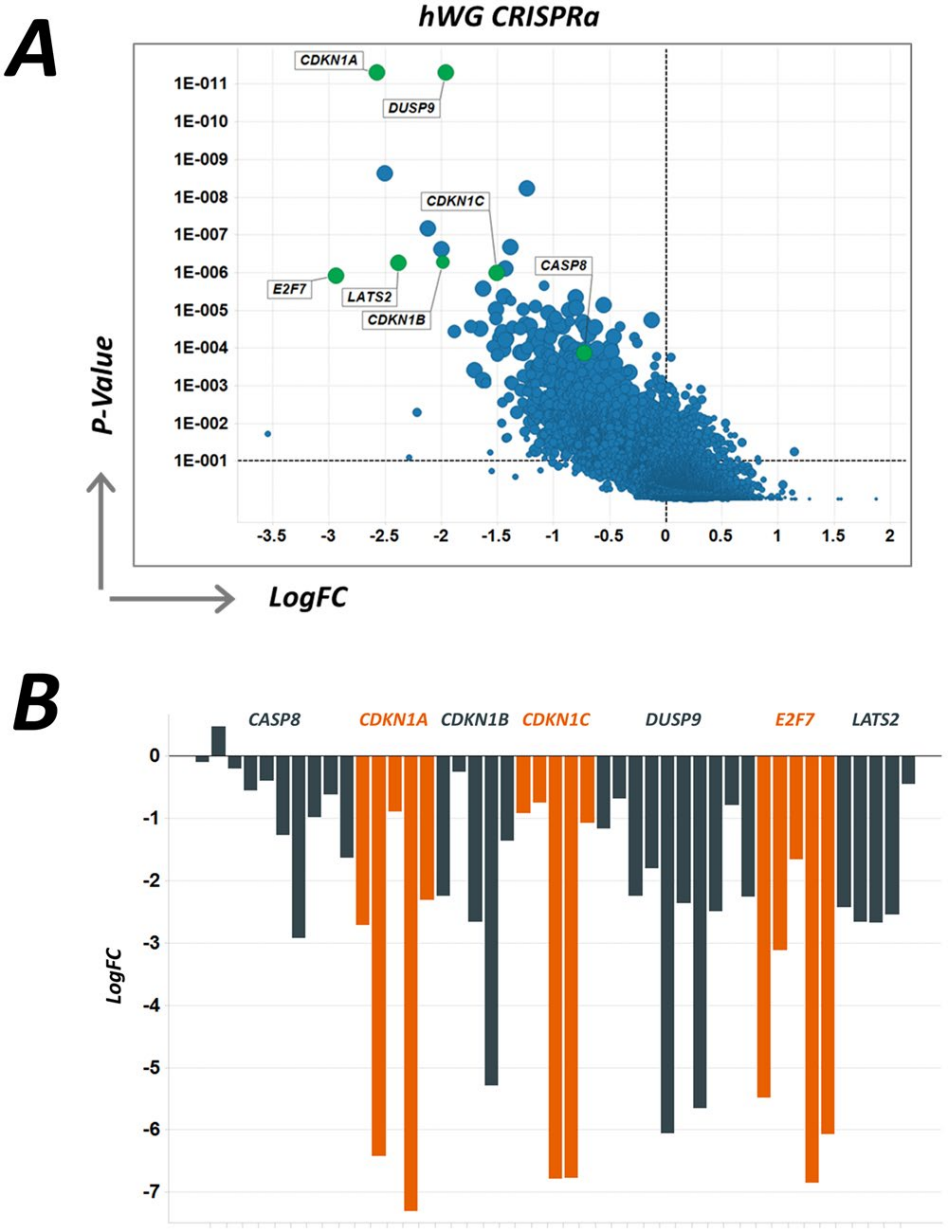
(**A**) Screen results showing drop-out rates from the CRISPRa screen and highlighting key hits, including cell cycle inhibitors (between the input sample (plasmid baseline) and end point control DMSO sample), plotting the magnitude of the effect (LogFC) against the MAGeCK RRA score (p-value). (**B**) Log2 depletion scores for each of the individual genes which showed drop out in the CRISPRa screen.

For the initial QC of the CRISPRa screen, we analysed sgRNAs that were lost (dropped out, Fig. 3A) during the course of the screen in the cells cultured in the absence of vemurafenib (DMSO control samples). Unsurprisingly, sgRNAs that bound to genes that negatively regulate the cell cycle, such as cyclin dependent kinase inhibitor 1 A (*CDKN1A*), *CDKN1B*, *CDKN1C* and *E2F7*, were lost from the cell population, along with guides targeting genes such as caspase 8 (*CASP8*), large tumour suppressor, Drosophila homologue 2 (*LATS2*), a serine/threonine kinase involved in cell cycle regulation, and dual specific phosphatase 9 (*DUSP9)*, a MAPK regulator (Fig. 3B).

**Figure 4:**
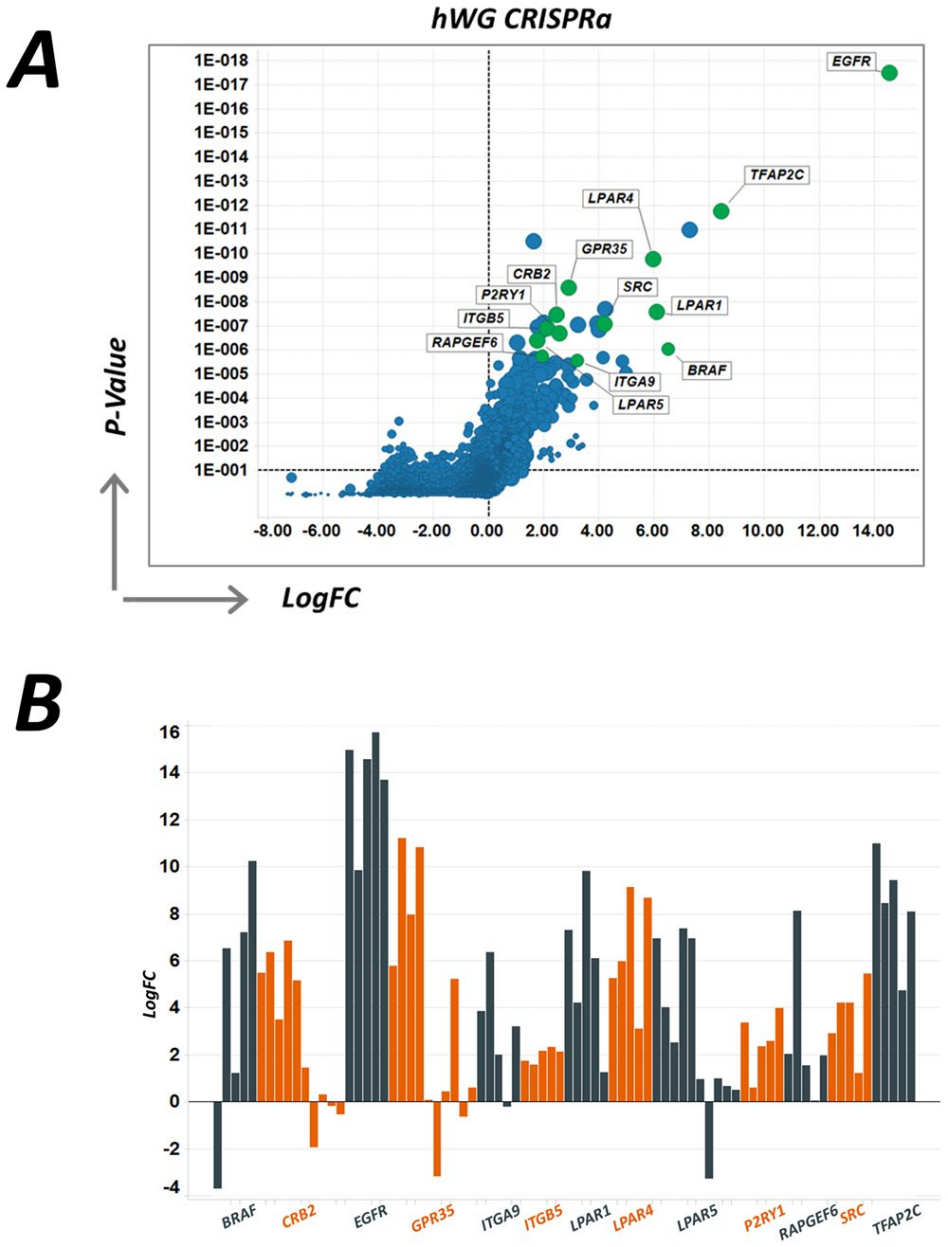
(**A**) Screen results for the enrichment of genes in the presence of vemurafenib and highlighting key hits, plotting the magnitude of the effect (LogFC) against the MAGeCK RRA score (p-value). (**B**) Log2 enrichment scores for each of the individual guides from hit genes in the CRISPRa screen.

Analyses of the sgRNAs enriched in the presence of vemurafenib identified a number of genes previously implicated in vemurafenib resistance by Konermann *et al*.^22^ (Fig. 4A,B), such as epidermal growth factor receptor (*EGFR*), several G protein coupled receptors (GPCRs) and *SRC*. All of these genes function to regulate ERK signalling, a known resistance mechanism to BRAF inhibition. In addition, and in contrast to previous studies, we also now identify BRAF itself as a gene that when overactivated drives resistance, as expected and supporting an increase in the overall screening sensitivity. As with the CRISPRi screen, we also identified a number of novel hits (Table 2), indicating there are additional pathways that could lead to vemurafenib resistance.

**Table 2:** CRISPRa hits for vemurafenib resistance.

GENE	GUIDES	RRA Score	p-value	FDR	RANK	Good guides	LogFC
*EGFR*	5	3.11E-18	2.64E-07	0.000825	1	5	14.097
*TFAP2C*	5	2.47E-12	2.64E-07	0.000825	2	5	8.4113
*FOXF1*	5	1.32E-11	2.64E-07	0.000825	3	5	7.5401
*IQSEC2*	10	8.51E-11	2.64E-07	0.000825	4	10	1.385
*LPAR4*	5	1.25E-10	2.64E-07	0.000825	5	5	5.9706
*GPR35*	10	2.29E-09	2.64E-07	0.000825	6	7	2.8763
*PAX2*	5	2.05E-08	7.93E-07	0.00165	7	5	4.1775
*LPAR1*	5	2.63E-08	7.93E-07	0.00165	8	5	5.9617
*CRB2*	10	2.79E-08	7.93E-07	0.00165	9	7	2.2622
*ONECUT3*	5	6.79E-08	1.32E-06	0.002166	10	5	1.8138
*SRC*	5	1.05E-07	1.59E-06	0.002166	11	5	4.1304
*EBF2*	5	1.34E-07	1.85E-06	0.002166	12	5	3.7063
*NFATC2*	10	1.36E-07	1.85E-06	0.002166	13	6	2.2585
*ITGB5*	5	1.38E-07	1.85E-06	0.002166	14	5	2.1651
*LPAR5*	10	1.49E-07	1.85E-06	0.002166	15	9	2.0537
*GATA5*	5	1.61E-07	1.85E-06	0.002166	16	5	3.8346
*P2RY1*	5	2.43E-07	2.38E-06	0.002621	17	5	2.4375
*TNNC1*	5	2.66E-07	2.64E-06	0.00275	18	5	1.7958
*GLIS3*	5	5.12E-07	2.91E-06	0.002866	19	5	3.1365
*BHLHE40*	5	7.70E-07	3.44E-06	0.003218	20	5	1.4929
*RUNX3*	10	1.17E-06	7.67E-06	0.006836	21	8	1.1072
*ITGA9*	5	1.57E-06	9.78E-06	0.007807	22	4	3.1412
*HES7*	5	1.66E-06	1.03E-05	0.007807	23	5	1.7808
*NEUROG3*	5	1.68E-06	1.03E-05	0.007807	24	5	1.4059
*RAPGEF6*	5	1.71E-06	1.08E-05	0.007807	25	5	1.9992

**Figure 5:**
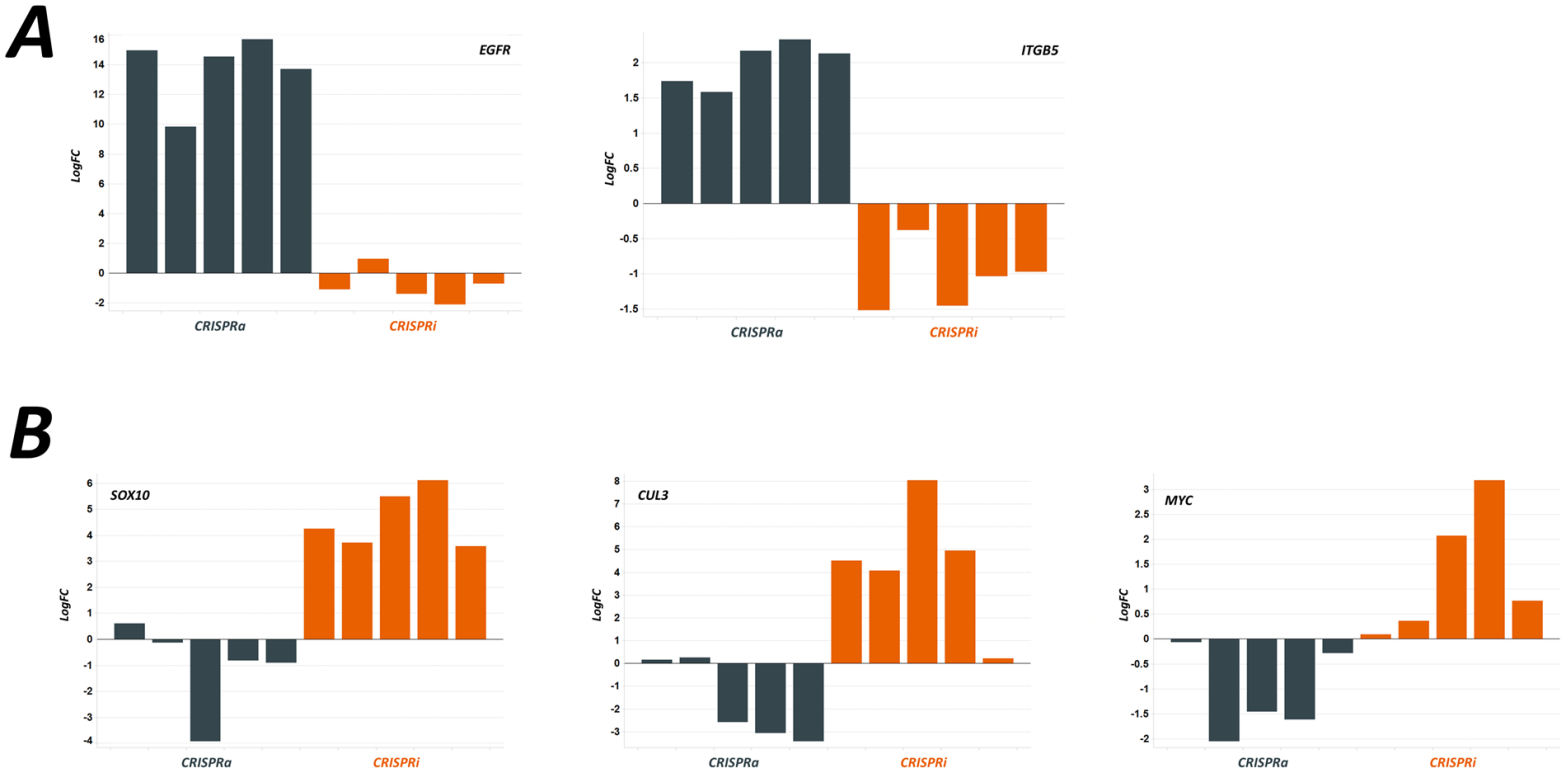
(**A**) Performance of individual guides from key hits which show enhanced resistance to vemerafenib when targeted by CRISPRa, plotted with the results from the CRISPRi screen which show opposing effects. (**B**) Genes which showed a resistance phenotype in CRISPRi, and opposing effects in the CRISPRa screen.

One of the advantages of directly comparable CRISPRi and CRISPRa data sets is the capacity for cross validation. For some of the genes shown to induce resistance in the CRISPRa screen, the guides targeting these genes dropped out to a degree in the CRISPRi screen. For example, sgRNAs targeting *EGFR* and integrin β5 (*ITGB5*), which resulted in vemurafenib resistance in the CRISPRa screen, dropped out in the CRISPRi screen in the presence of the drug (Fig. 5A). Similarly, genes that are a hit in the CRISPRi screen (i.e. suppression of their transcription leads to drug resistance) are lost from the CRISPRa screen, as their expression induces sensitivity to vemurafenib. For example, sgRNAs targeting *SOX10*, cullin 3 (*CUL3*) and MYC, dropped out in the CRISPRa screen in the presence of vemurafenib and were enriched in the CRISPRi screen (Fig. 5B).

**Figure 6:**
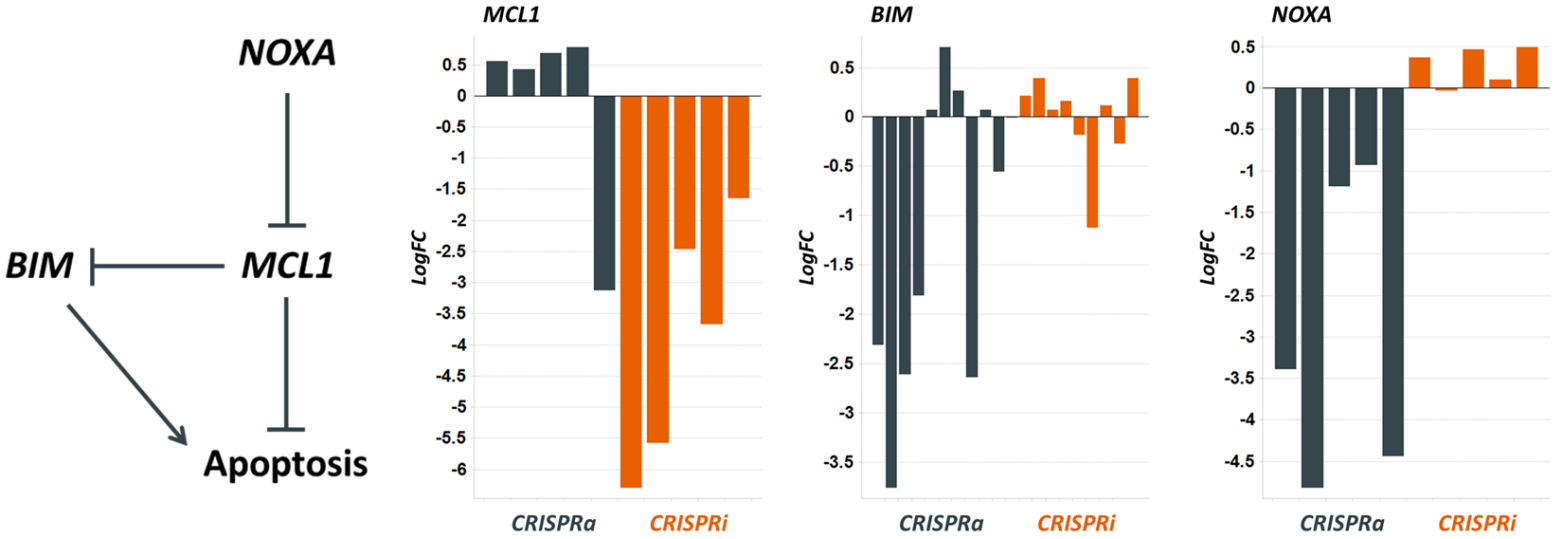
Three genes involved in the control of apoptosis, and their performance in each of the CRISPRi and CRISPRa screens. Inhibition of MCL1 by CRISPRi greatly enhanced cell death whilst activation by CRISPRa partially abrogates the effect in response to drug-treatment. Opposing effects were observed for the MCL1 regulators BIM and NOXA, in line with their effect in inhibiting apoptosis.

In addition to providing a compelling strategy for the validation of drug resistance targets, the parallel analysis of CRISPRi and CRISPRa data sets also allows for the identification of pathway modulators that affect drug both sensitivity and resistance, but in which sgRNAs are not reciprocally lost or increased in each screen. In particular, we found that sgRNAs targeting the anti-apoptotic gene *MCL1* dropped out substantially in the CRISPRi screen (Fig. 6), but were not enriched in the CRISPRa screen. However, sgRNAs targeting two regulators of MCL-1, NOXA and BIM, were lost in the CRISPRa screen, but had no discernible effect in the CRISPRi screen (Fig. 6). Similar losses of the sgRNAs targeting *MCL1*, *BCL2L11* (the gene encoding BIM) and *PMAIP1* (the gene encoding NOXA) were not evident in the DMSO control, indicating that this apoptotic pathway is important in the response to vemurafenib and is not affected in the screens simply because these proteins regulate cell viability (Figure S2). Thus, the control of apoptosis triggered by drug treatment can be faithfully monitored by reciprocal analysis of the paired dataset to yield a greater resolution dataset to describe the mechanism of drug-induced cell death.

## Discussion

We have compared data from a CRISPRko screen examining resistance to vemurafenib with CRISPRi and CRISPRa screens with the same drug in the same cell type (the A375 melanoma cell line). The CRISPRko and CRISPRi data overlap substantially, with the same hits being identified in both screens and these are consistent with published data sets^28^. Moreover, our data indicate that with a modified tracrRNA, CRISPRi might be a more sensitive screening platform for some targets than an unmodified CRISPRko screening tool. A major consideration in this regard is the identity of the tracrRNA used in either screen. We have previously published a direct side by side comparison of two sequences and shown the advantages of a modified system which more closely models the endogenous complex^29^. The data presented here appear to underline that observation and are in agreement with other published studies showing the advantages of this approach for CRISPRko^30^, and those which highlight the effect in CRISPRi^31^. Our CRISPRa data set is also in good agreement with a similar published study^5,22^, and, for some targets, allows cross validation of hits identified from the CRISPRi screen. This was evident for *EGFR* and *IGTB5*, which induce resistance when overexpressed in CRISPRa screens and loss of the expression of these genes leads to increased sensitivity to vemurafenib. Conversely, loss of expression of *SOX10*, *CUL3* and *MYC* leads to drug resistance in a CRISPRi screen and overexpression of these genes is associated with vemurafenib sensitivity in the CRISPRa screen. These data indicate that running CRISPRa and CRISPRi screens in parallel provides an exceptionally powerful data set for target identification and should speed up the validation process.

That CRISPRko and CRISPRi data sets provided a highly similar hit list indicates that CRISPRi might overcome some of the shortcomings of CRISPRko screens. For example, CRISPRko isn’t ideally suited to the study of hypomorphic phenotypes, including that of essential genes, and complete gene knock out might not be the best model for identifying targets that are druggable, as drugs are likely to lead to a reduction in function of a target, but not its complete absence. Another chink in the armour of CRISPRko is the application to amplified loci, where multiple cuts can cause an off-target DNA damage response^8,9^. As dCas9 does not alter the sequence of genomic DNA, it could be used to address which genes are important in amplified regions of chromosomes or to investigate levels of repression needed to target proteins encoded in double minute chromosomes. Moreover, the activity of dCas9 can be engineered to be regulatable as its effect in concert with sgRNAs in a CRISPRi or CRISPRa screen is reversible, which is not the case for the Cas9 nuclease which cuts the DNA leading to irreversible modification. CRISPRi and CRISPRa approaches will also be more suitable for studying differential expression of long noncoding RNAs, genes that have proven difficult to target effectively with the CRISPR knockout platform. Finally and unique to CRISPRa is the prospect to study gain-of-function phenotypes – this opens up myriad new paths for novel drug discovery.

Overall, our data indicate that CRISPRi and CRISPRa are valuable additional new CRISPR screening tools for target identification and validation. Dual directional approaches such as ours now provide the new opportunity to achieve increased depth of hit finding in the analysis and offer novel discovery pathways by exploring opposing function and more complete gene network perturbation. This phenomenon has been robustly corroborated by the mechanistic de-orphaning of the drug rigosertib^32^ and has particular application to drug mechanism of action analysis. Importantly, with appropriate design, the power of enrichment-based screening (e.g. resistance screening) can now be co-opted to identify genes which result in sensitisation by analysing the effect of the opposing function. Thus, whilst the effect of depletion of a target gene on diminished cell viability might be hard to study with a loss-of-function screen, the response of cells to hyper-activating this component on overcoming cell death can be readily detected, providing valuable genetic insights into cellular physiology. All three approaches, CRISPRko, CRISPRi and CRISPRa, have their independent strengths and limitations, many of which will become more apparent as these techniques become more widely used. However it appears clear that the high precision and penetrance of a knock out approach is greatly enhanced when combined with datasets produced by both CRISPRi and CRISPRa.

## Methods

### Cell lines

A375 melanoma cells (ATCC, USA) were thawed and cultured in DMEM supplemented with 10% FBS and 2mM L-glutamine (all supplied by Gibco, UK). Cells were routinely checked for mycoplasma and identity verified by STR analysis.

### Library generation

We generated two pooled whole genome guide RNA libraries using the guide RNA design algorithms used by Horlbeck *et al*.^24^, for use in CRISPRi and CRISPRa screens. Each sgRNA was synthesised (Custom Array Inc) to target the transcriptional start site (CRISPRa library) or downstream of the TSS (CRISPRi library). In the CRISPRi library, a modified tracrRNA sequence, as described in Chen *et al*.^31^ and Cross *et al*.^29^, (5′-GTTTAAGAGCTA*TGCTG*GAAA*CAGCA*TAGCAAGTT-3′) was used. An all-in-one lentivirus plasmid vector was built for the CRISPRi screen comprising a selection marker (puromycin resistance), the expression cassette for dCas9 fused to the KRAB repressor domain and the sgRNA sequence. This backbone is termed HZD044_pLentiCRISPRi. For the CRISPRa screen, we had 3 vectors synthesized following the information published by Konnerman *et al*.^22^. For the CRISPRko screen we used the second generation guides used by the Zhang laboratory^27^ (6 sgRNAs per target gene). The tracrRNA sequence used in the original GeCKOv2 library (5- GTTTTAGAGCTAGAAATAGCAAGTTAAAATAAGGC-3^27^) was used in the CRISPRko screen.

Each pooled sgRNA library was cloned into the relevant vector backbone using a Gibson Assembly Master Mix kit (New England BioLabs, NEB #E2611S/L) in accordance with the manufacturer’s instructions. Library plasmids were purified using a Qiagen Plasmid Plus purification system in accordance with the manufacturer’s instructions.

### Lentivirus production

HEK293T cells (ATCC, USA) were grown in DMEM and 10% FBS (Gibco, UK). 24 hours ahead of transfection with the library vectors, HEK293T cells were seeded into T225 flasks (Corning) at 40% confluency. The following day, the cells were transfected with the library plasmids using Lipofectamine 3000 (Invitrogen, USA) and Virapower packaging virus (LifeTechnologies, UK) in accordance with the manufacturer’s instructions. Briefly, the medium was removed from the HEK293T cells and the DNA–lipid mix was added to the cells in Optimem medium (Gibco, UK) and left for 6 hours, after which the transfection mix was removed and replaced with DMEM containing 10% FBS and 1% BSA (Sigma-Aldrich). The medium was harvested 48 hours later and centrifuged at 500×g for 10 min at 4 °C. The virus was further concentrated using Lenti-X concentrator (Clontech #631232) in accordance with the manufacturer’s instructions. The viral supernatant was aliquoted and stored at −80 °C in DMEM with 10% FBS and 1% BSA.

### Cell transduction and screening protocol

Functional titration was used to identify the transduction conditions that allow use of a low MOI (~0.3) at which the majority of cells are infected with a single viral particle. This is particularly important for screening where the generation of single knockouts is the main goal. Once each lentiviral library had been functionally titrated in A375 cells, the cells were trypsinized, seeded in complete medium supplemented with 8 μg/ml polybrene (Sigma-Aldrich) and transduced with each library according to standard lentiviral transduction protocols. Briefly, for the CRISPRko and CRISPRi libraries, cells were seeded into 12 well dishes at 2 × 10^6^ cells per well and spinfected for 2 hours at 2000rpm at 37 °C using virus diluted to achieve an MOI of 0.3. A T175 cm^2^ flask of untransfected cells was also set up for each cell line. In all screens, at least 1 × 10^8^ cells were transduced in total. The cells from all spinfected wells were resuspended, transferred to a 50 ml falcon and centrifuged at 1000 rpm for 5 minutes. The supernatant was removed and cells were resuspended in 50 ml fresh media (without polybrene) and transferred to T175 cm^2^ flasks at 12 × 10^6^ cells per flask per library per line. 48 hours later the transduced and non-transduced cells were treated with puromycin at a final concentration of 0.5 μg/ml. 72 hours after addition of puromycin, 3 technical replicate pellets of 74 × 10^6^ cells were harvested and flash frozen from each cell line transduced with each library (T_1_). The remaining cells were maintained in puromycin in 5 layer flasks (Falcon) at 12 × 10^6^ cells per flask. The cells were counted and reseeded at 12 × 10^6^ per 5 layer flask every 3–4 days and puromycin selection was stopped four days after spinfection when all non-transduced cells were dead.

For the CRISPRa screen functional titration was used to identify the transduction conditions that allow use of a high, non-toxic, MOI for both dCas9-VP64 (MOI ~0.8) and MS2-P65-HSF1 (MOI ~0.4) components. For the CRISPRa library, similar to CRISPRi library, we identified the transduction conditions that allow use of a low MOI (~0.3). First, A375 cells were transduced with dCas9-VP64 lentivirus, expression of which was selected by blasticidin (2.5 ug/ml) selection. This dCas9-VP64 population was then transduced with the MS2-P65-HSF1 transcriptional activator component, whose expression was selected for by hygromycin B (200 ug/ml). Finally, the double-stable cell population was transduced with the CRISPRa library (selection marker: zeocin (300 ug/ml)). A minimum of 4 × 10^7^ viable cells were maintained at all times throughout the experiments and in all treatment conditions.

For all the screens cells were passaged for a total of 16 days and final pellets were harvested 16 doublings (T_2_) after T_1_. All frozen pellets were thawed and gDNA extracted using Qiagen Blood Maxi kit. DNA concentration was determined using a Nanodrop spectrophotometer and at least 230 µg of genomic DNA for each sample was then amplified with PCR to generate amplicons of the sgRNA cassette^28^ using a forward primer: TCGTCGGCAGCGTCAGATGTGTATAAGAGACAGU–[Variable]–TGTGGAAAGGACGAAACACC; and a reverse primer: GTCTCGTGGGCTCGGAGATGTGTATAAGAGACAGGATCAATTGCCGACCCCTCC. These amplicon samples were purified using Agencourt beads (Beckman) and deep sequenced on an Illumina NextSeq platform/system (Microsynth AG, Switzerland).

### Data analysis

Raw NGS libraries were evaluated for quality using FASTQC version 0.11.5. (Babraham Institute, Cambridge UK). Guide counts were obtained using an in-house customized version of the MAGeCK workflow version 0.5.5, which took into account guide staggering from the experimental protocol. Briefly, guides were trimmed and mapped with exact string counts from each file to provide raw counts for each guide found in the library. Guide counts were normalised within each group (median-based) and Log_2_ fold change (LogFC) was calculated to determine the change in abundance of each guide in each sample. RRA values (p-values) were determined using the MAGeCK algorithm (version 0.5.5), as described in Li *et al*.^6^. Unless otherwise indicated, LogFC is determined between the early timepoint (T_1_) and 16 population doublings (T_2_).

### Data availablity

The datasets generated during the current study are available from the corresponding author on reasonable request.

## Electronic supplementary material

Supplementary Information
