# Supplementary material for: Dual direction CRISPR transcriptional regulation screening uncovers gene networks driving drug resistance
Source: Sci Rep. 2017 Dec 18;7:17693. doi: 10.1038/s41598-017-18172-6 (PMC5735189; doi:10.1038/s41598-017-18172-6)
Supplement: Supplementary file 1 — Supplementary Information [file 41598_2017_18172_MOESM1_ESM.pdf]

SUPPLEMENTARY INFORMATION FOR:

Dual direction CRISPR transcriptional regulation screening uncovers gene networks driving drug resistance

Carlos le Sage, Steffen Lawo, Prince Panicker, Tim M.E. Scales, Syed Asad Rahman, Annette S. Little, Nicola J. McCarthy, Jonathan D. Moore and Benedict C.S. Cross*.

Horizon Discovery, 8100 Cambridge Research Park, Waterbeach, Cambridge, CB25 9TL, United Kingdom.

*Author correspondence to Benedict C.S. Cross (Benedict.Cross@horizondiscovery.com)

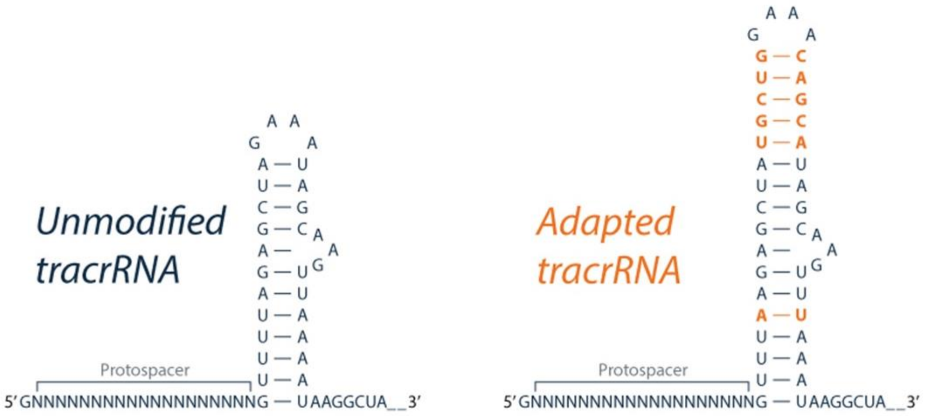

Figure S1 | Schematic showing the adapted tracrRNA sequence used in the CRISPRi screen.

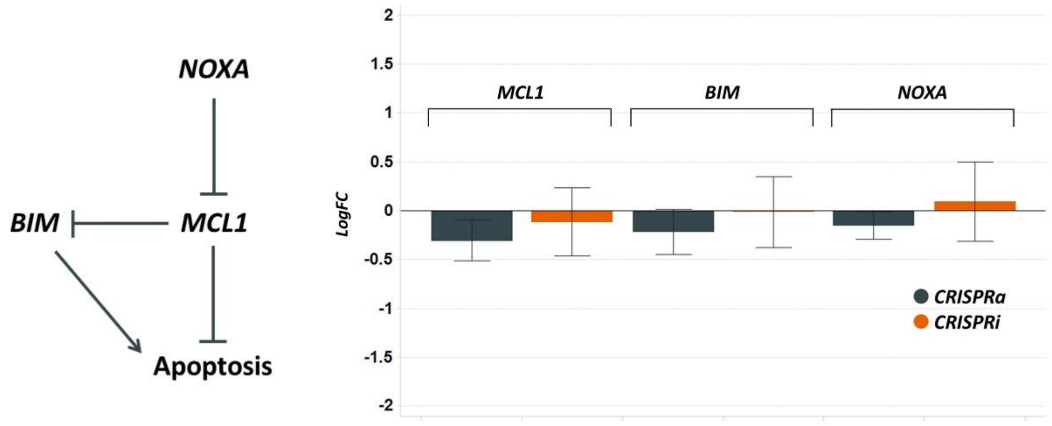

Figure S2 | Gene level abundance changes of the MCL1, BIM and NOXA from the baseline to endpoint (DMSO) in the CRISPR screens indicating that these components do not directly appear to affect viability in the absence of drug-treatment.
